# Analysis of brain networks and fecal metabolites reveals brain–gut alterations in premenopausal females with irritable bowel syndrome

**DOI:** 10.1038/s41398-020-01071-2

**Published:** 2020-11-02

**Authors:** Vadim Osadchiy, Emeran A. Mayer, Kan Gao, Jennifer S. Labus, Bruce Naliboff, Kirsten Tillisch, Lin Chang, Jonathan P. Jacobs, Elaine Y. Hsiao, Arpana Gupta

**Affiliations:** 1 G. Oppenheimer Center for Neurobiology of Stress and Resilience, University of California, Los Angeles, Los Angeles, CA USA; 2 David Geffen School of Medicine, University of California, Los Angeles, Los Angeles, CA USA; 3 Department of Urology, David Geffen School of Medicine, University of California, Los Angeles, Los Angeles, CA USA; 4 Vatche and Tamar Manoukian Division of Digestive Diseases, VA Greater Los Angeles Healthcare System, Los Angeles, CA USA; 5 UCLA Microbiome Center, VA Greater Los Angeles Healthcare System, Los Angeles, CA USA; 6 Division of Gastroenterology, Hepatology and Parenteral Nutrition, VA Greater Los Angeles Healthcare System, Los Angeles, CA USA; 7 Department of Integrative Biology and Physiology, University of California, Los Angeles, Los Angeles, CA USA

**Keywords:** Physiology, Pathogenesis, Neuroscience

## Abstract

Alterations in brain–gut–microbiome (BGM) interactions have been implicated in the pathogenesis of irritable bowel syndrome (IBS). Here, we apply a systems biology approach, leveraging neuroimaging and fecal metabolite data, to characterize BGM interactions that are driving IBS pathophysiology. Fecal samples and resting state fMRI images were obtained from 138 female subjects (99 IBS, 39 healthy controls (HCs)). Partial least-squares discriminant analysis (PLS-DA) was conducted to explore group differences, and partial correlation analysis explored significantly changed metabolites and neuroimaging data. All correlational tests were performed controlling for age, body mass index, and diet; results are reported after FDR correction, with *q* < 0.05 as significant. Compared to HCs, IBS showed increased connectivity of the putamen with regions of the default mode and somatosensory networks. Metabolite pathways involved in nucleic acid and amino acid metabolism differentiated the two groups. Only a subset of metabolites, primarily amino acids, were associated with IBS-specific brain changes, including tryptophan, glutamate, and histidine. Histidine was the only metabolite positively associated with both IBS-specific alterations in brain connectivity. Our findings suggest a role for several amino acid metabolites in modulating brain function in IBS. These metabolites may alter brain connectivity directly, by crossing the blood–brain-barrier, or indirectly through peripheral mechanisms. This is the first study to integrate both neuroimaging and fecal metabolite data supporting the BGM model of IBS, building the foundation for future mechanistic studies on the influence of gut microbial metabolites on brain function in IBS.

## Introduction

Alterations in brain–gut–microbiome (BGM) interactions have been implicated in the pathogenesis of irritable bowel syndrome (IBS)^1^. However, despite a growing body of preclinical and clinical studies, the precise mechanisms by which these interactions contribute to IBS symptom generation remains incompletely understood.

Neuroimaging studies have previously demonstrated structural and functional differences between IBS patients and healthy controls (HCs), especially in the default mode (DMN), somatosensory, and salience networks^2^. One of the most consistent findings in IBS and other disorders of visceral hypersensitivity and chronic pain has been alterations in the structure and function of key regions of the somatosensory network, including the basal ganglia (composed of the caudate, putamen, and globus pallidus)^2^. The basal ganglia, due in part to the structure’s many cortical and thalamic afferent and cortical efferent connections, is responsible for central processing and modulation of both visceral and somatic nociception^3,4^. As females tend to experience more severe and frequent IBS symptoms than males, it is unsurprising that many of the described brain network alterations are sex-specific^2,5^.

We have previously provided evidence supporting a role for spore forming gut bacteria of the order *Clostridiales* in contributing to alterations in evoked visceral hypersensitivity through microbial modulation of both cortical and subcortical brain regions, including the basal ganglia^6^. Although this work and many others reveal insights into the complex relationship between gut microbes and IBS, there remains insufficient granularity for robust mechanistic studies to take place. Microbes may modulate BGM interactions by directly activating vagal afferents or by stimulating dendritic cells, thereby triggering an immune-mediated signaling cascade to the central nervous system (CNS) without the help of any small molecule intermediates^7^.

However, it is perhaps more likely that gut microbiota-derived or microbiota-modified metabolites play a role in driving clinically meaningful changes in the BGM axis^8^. Tryptophan and related metabolites have been the most extensively studied within this context^9^, with previously described alterations in levels of blood tryptophan^10^ and immediate downstream products^11,12^ in patients with IBS, compared with HCs. Recent evidence also suggests that histamine, derived from the amino acid histidine, may play an important role in IBS pathophysiology and symptomatology^13^. The H1R, H2R, and H4R gut histamine receptors mediate sensorineural signaling^14^, immune activation^15^, and nociception^16^, respectively—representing key avenues for gut–brain communication^17^. Histamine may also act centrally, given that the basal ganglia is densely populated by histamine receptors, with a large body of evidence to suggest that histamine can directly influence basal ganglia output^18^.

In this cross-sectional study, we integrate fecal metabolite and neuroimaging data to explore the relationship between BGM alterations in IBS females. Although results from cross-sectional studies do not allows us to make inferences about causality, we hypothesize that amino acid metabolites are playing a key role in driving some of the neuroimaging changes in IBS. These results will allow for novel hypothesis generation and will serve as the foundation for future, mechanistic studies.

## Methods

### Subjects

138 right-handed adult premenopausal females were recruited (99 female IBS patients and 39 female HCs) provided a stool sample and underwent multimodal brain-imaging studies at UCLA. All MRI scans were taken during the follicular phase of the menstrual cycle, with stool collections typically occurring within a week of the scan. Subjects were all premenopausal as determined by medical history. IBS subjects met Rome III symptom criteria for IBS^19^. A gastroenterologist or nurse practitioner with expertise in IBS obtained a medical history and physical exam to confirm the IBS diagnosis. IBS patients with any bowel habit were included.

Exclusionary criteria for all subjects included (1) serious medical conditions or were taking medications which could compromise interpretation of the brain imaging; (2) ongoing major psychiatric diagnoses or use of psychotropic medications in the past 6 months (subjects were not excluded for lifetime incidence of psychiatric disorders or for intake of low-dose tricyclic antidepressants for non-psychiatric indications); (3) use of antibiotics in the past 3 months, selective serotonin reuptake inhibitors, opioids; and (4) excessive physical exercise (e.g., marathon runners).

### Ethics and statement of informed consent

All procedures complied with the principles of the Declaration of Helsinki. Informed consent was obtained from all participants. This study was approved by the Institutional Review Board (12-001802, 16-000187, 15-001591) at the University of California, Los Angeles.

### Diet questionnaire

Diet was assessed using through self-reported questionnaire, where participants were asked to select which diet is consumed on a regular basis. Options included: Standard American (characterized by high consumption of processed, frozen, and packaged foods, pasta and breads, and red meat; vegetables and fruits are not consumed in large quantities), Modified American (high consumption of whole grains including some processed, frozen, and packaged foods; red meat is consumed in limited quantities; vegetables and fruit are consumed in moderate to large quantities), Mediterranean (high consumption of fruits, vegetables, beans, nuts, and seeds; olive oil is the key monounsaturated fat source; dairy products, fish, and poultry are consumed in low to moderate amounts and little red meat is eaten), and all other diets that do not fit into the above categories.

### Fecal metabolomics

Fecal samples were stored at −80 °C and shipped to Metabolon (Durham, NC) for processing and analysis as a single batch on their global metabolomics and bioinformatics platform using ultrahigh performance liquid chromatography and tandem mass spectrometry^20^. Raw data was curated by mass spectrometry using specialized software as previously described^20^. The number of missing data was low (<3%); missing values of raw data were filled up by median value, and ineffective peaks were removed through the interquartile range denoising method. In addition, the internal standard normalization method was employed in the data analysis. The dataset for the multiple classification analysis was compiled from the metabolite profiling results and a 3D matrix involving metabolite numbers, sample names, and normalized peak intensities were fed into the MetaboAnlyst web software 3.0 (http://www.metaboanalyst.ca)^21^.

### Resting state brain connectivity

#### Magnetic resonance imaging acquisition

All patients underwent an imaging session in a 3 T Siemens Allegra MRI Scanner (Siemens, Erlangen, Germany) for a high-resolution T1 structural scan, and a resting-state functional scan. Whole-brain structural and functional (resting-state) imaging data was acquired using the following parameters: acquisition parameters for high-resolution T1-weighted images were as follows: echo time/repetition time (TE/TR) = 2.85/2200 ms, inversion time = 750 ms, field of view (FOV) = 256 mm, slice thickness = 1 mm, 176 slices, 256 × 240 acquisition matrix, voxel size = 1 mm^3^. Functional resting-state scans were acquired with eyes closed and an echo-planar sequence with the following parameters: TE/TR: 28/2000 ms, flip angle = 77°, scan duration = 10 min, FOV = 220 mm, slices = 300, slice thickness = 4.0 mm, and slices were obtained with whole brain coverage.

#### MRI processing

##### Structural preprocessing

Preprocessing and quality control of structural images were done using Statistical Parametric Mapping 12 (SPM12)^4^. All structural images were skull stripped, segmented, then normalized to the MNI T1 template. This created normalized T1 images for every subject along with segmented images (gray matter, white matter, and cerebrospinal fluid (CSF)) in normalized space.

#### Structural processing

##### Structural image parcellation

T1-image segmentation and cortical and subcortical regional parcellation were conducted using FreeSurfer v.6.0^5–7^ following the nomenclature described in the Destrieux and Harvard-Oxford subcortical atlas^8,9^. The parcellation results in the labeling of 165 cortical regions, 74 bilateral cortical structures, 7 subcortical structures, the midbrain, and the cerebellum.

##### Resting-state fMRI preprocessing

Preprocessing and quality control of functional images was done using SPM-12 software (Welcome Department of Cognitive Neurology, London, UK) and involved slice–time correction and motion correction for the six realignment parameters. If any motion was detected above 2 mm translation or 2˚ rotation, the scan, along with the paired structural scan was discarded. In order to robustly take account the effects of motion, root mean squared (RMS) realignment estimates were calculated as robust measures of motion using publicly available MATLAB code from GitHub. The resting state images were then co-registered to their respective anatomical T1 images. Each T1 image was then segmented and normalized to a smoothed template brain in Montreal Neurological Institute (MNI) template space. Each subject’s T1 normalization parameters were then applied to that subject’s resting state image, resulting in an MNI space normalized resting state image. The resulting images were smoothed with 5 mm^3^ Gaussian kernel. For each subject, a sample of the volumes was inspected for any artifacts and anomalies. Levels of signal dropout were also visually inspected for excessive dropout in a priori regions of interest.

#### Resting-state fMRI processing

##### Functional network construction

Functional brain networks were constructed using the CONN 17 toolbox^10^ in MATLAB. Regions from the Destrieux and Harvard-Oxford Subcortical Atlases were entered as ROIs^8,9^. To summarize, all pre-processed, normalized images were first corrected for noise using the CompCor method to remove physiological noise without regressing out the global signal^22^. Confounds for the six motion parameters along with their first-order temporal derivatives, along with confounds emerging from white matter and CSF, and first-order temporal derivatives of motion, and RMS were removed using regression. The images were then band-passed filtered between 0.01/s < *f* < 0.1/s to reduce low-frequency and high-frequency noise that are not indicative of intrinsic brain activity. Linear measures of ROI-to-ROI functional connectivity were computed using Fisher-transformed correlations representing the association between average temporal BOLD time series signals across all voxels in a brain region. The final outputs for each subject consisted of a 165 × 165 matrix consisting of Fisher-transformed *Z* correlation values between each ROI.

### Partial least-squares discriminant analysis (PLS-DA)

PLS-DA was conducted in R (Boston, MA) to explore the difference between groups by incorporating known classifications for the metabolites and for whole brain for the resting state connectivity. PLS-DA was ran separately for brain and metabolites. In order to prevent overfitting of the model with PLS-DA, we ran permutation tests as previously published^23,24^. The metabolites and brain connectivity regions with values of the first PLS-DA component of variable importance projection (VIP) in PLS-DA >1.0 were assessed by Student’s *t*-test controlling for age, body mass index (BMI), and diet. *P-*values were adjusted with the Benjamini–Hochberg false discovery rate (FDR) procedure^25^. FDR-corrected *P* values, which referred to as *q* values were reported. The metabolite and brain connectivity with VIPs > 1.0 and *q* values <0.05 was selected as significantly different between the two groups. For the metabolites the FC was also calculated to investigate the difference by comparing the mean value of the peak area obtained between the two groups. The KEGG pathway databases for *Homo sapiens* in the MetaboAnalyst 3.0 were used to explore metabolic impact pathways^21^. The pathway with a *q* value <0.05 and an impact value >0.05 was defined as a significant impact pathway.

### Correlation analysis

The partial correlation analysis between significantly changed metabolites and resting-state connectivity controlling for age, BMI, and diet, was conducted using SPSS software version 21 (SPSS Inc., Chicago, IL, USA). The relations between metabolites and resting-state function with *P* value < 0.05 were considered as significantly correlated. Means and standard deviations (SD) are presented for normally distributed data. Medians and IQR are reported for non-normally distributed data.

## Results

### Clinical measures

In total, 138 female subjects participated in this study (99 IBS, 39 HCs). Ages ranged from 18 to 44 (IBS: median = 24, interquartile ranges [IQR] = [21, 29]); HC: median = 27, IQR = [20, 34]; *p* = 0.357). BMI ranged from 18.4 to 40.2 (IBS: mean = 23.5, SD = 3.94; HC: mean = 24.0, SD = 2.74; *p* = 0.415). With respect to bowel habit, 36 subjects reported constipation-predominant IBS, 28 reported diarrhea-predominant, 26 reported mixed, and 9 were unspecified.

For HCs, 64% of females reported a standard American diet, 5% a modified American diet, 5% a Mediteranian diet, and 26% a diet in the “other” category. For IBS, 53% of females reported a standard American diet, 12% a modified American diet, 1% a mediteranian diet, and 34% a diet in the “other” category.

### Significant metabolites differences between IBS and HC females

**Fig. 1:**
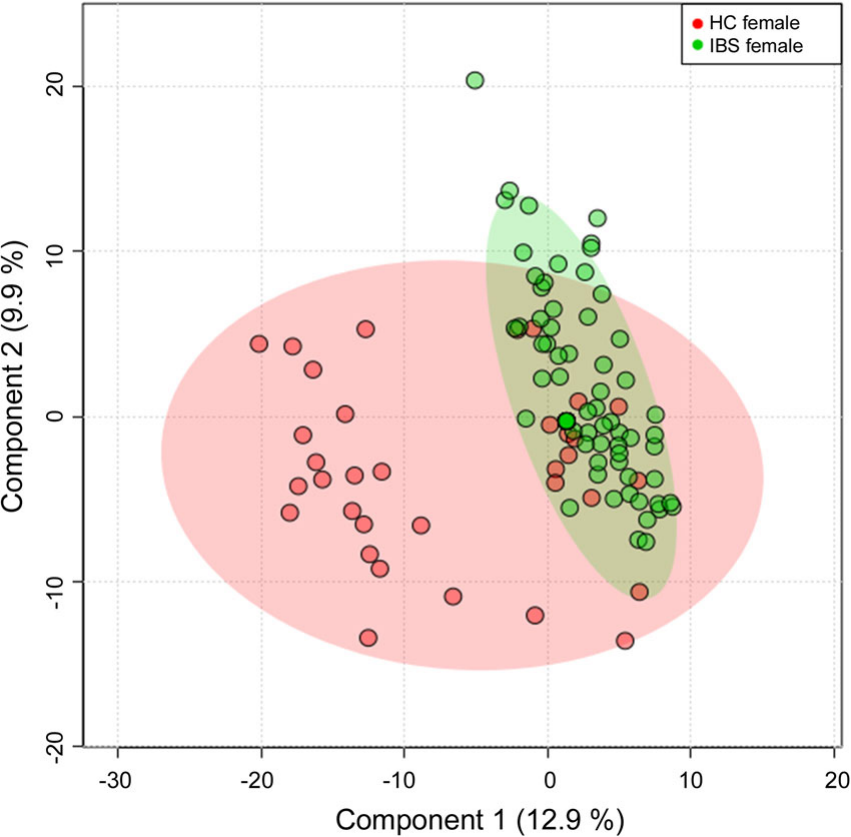
Multivariate analysis in the fecal samples for human subjects. The PLS-DA score map in the fecal samples between HC and IBS females.

A total of 1154 metabolites were identified by metabolomic profiling. Classifications were found in the PLS-DA (Fig. 1). A total of 125 significant differences in metabolites (VIP > 1.0 and *q* value < 0.05) were identified between premenopausal IBS and HCs, including 32 amino acid metabolites, 15 carbohydrate metabolites, 5 cofactors, and vitamins metabolites, 2 energy metabolites, 35 lipid metabolites, 17 nucleotide metabolites, 7 peptide metabolites, and 12 xenobiotic compounds (Table S1). In detail, compared with HC females, 43 metabolites were significantly lower in IBS, particularly lipids, such as N-palmitoyl-sphinganine (d18:0/16:0) (fold change [FC] = 0.14, *q* = 1.7E−09), palmitoyl-oleoyl-glycerol (16:0/18:1) (FC = 0.12, *q* = 1.1E−07), and oleoyl-oleoyl-glycerol (18:1/18:1) (FC = 0.19, *q* = 7.11167E−07), while 82 metabolites were significantly higher in IBS females, such as gamma-glutamylleucine (FC = 1.57, *q* = 8.44067E−11), 2-hydroxyglutarate (FC = 1.47, *q* = 1.2E−09), and N-acetylmethionine sulfoxide (FC = 1.73, *q* = 7.2E−08).

### Significantly impacted pathways between IBS and HC females

A total of 51 pathways were identified that differed between IBS and HC females in KEGG database for *H. sapiens* (Supplementary information Table S2). In detail, four pathways including aminoacyl-tRNA biosynthesis (*q* = 0.002, impact value = 0.11268), pyrimidine metabolism (*q* = 0.005, impact value = 0.22819), alanine, aspartate, and glutamate metabolism (*q* = 0.02, impact value = 0.23362), and beta-alanine metabolism (*q* = 0.03, impact value = 0.07744) were identified as significant impact pathways (Table 1).

**Table 1:** Significantly differentially abundant pathways between HC and IBS females.

Pathways	Total	Expected	Hits	*p* value	−Log(*p*)	*q* value^a^	Impact
Aminoacyl-tRNA biosynthesis	75	2.6485	11	4.28E−05	10.058	0.002184	0.11268
Pyrimidine metabolism	60	2.1188	9	0.0001886	8.5759	0.004810	0.22819
Alanine, aspartate, and glutamate metabolism	24	0.84753	5	0.001221	6.7081	0.020757	0.23362
Beta-alanine metabolism	28	0.98878	5	0.002526	5.9812	0.032203	0.07744
Glutathione metabolism	38	1.3419	5	0.009777	4.6278	0.092727	0.04893
Nitrogen metabolism	39	1.3772	5	0.010909	4.5182	0.092727	0.00000
Valine, leucine, and isoleucine biosynthesis	27	0.95347	4	0.013666	4.2928	0.099567	0.11553
Histidine metabolism	44	1.5538	5	0.017928	4.0214	0.111191	0.14053
Phenylalanine metabolism	45	1.5891	5	0.019622	3.9311	0.111191	0.05939
Glycine, serine, and threonine metabolism	48	1.6951	5	0.025326	3.6759	0.129163	0.32425
Ubiquinone and other terpenoid-quinone biosynthesis	36	1.2713	4	0.036090	3.3217	0.167326	0.06488
Purine metabolism	92	3.2489	7	0.041258	3.1879	0.175347	0.06907

^a^*p* value was calculated with FDR correction.

### Significant differences in resting-state connectivity between IBS and HC females

The differences in resting-state connectivity between IBS and HCs were evaluated (Table 2). Compared with HCs, IBS had significantly higher resting-state connectivity, including left DMN (dorsal part of the posterior cingulate gyrus) to left basal ganglia (putamen) and right somatosensory network (superior frontal gyrus) to right basal ganglia (putamen), both *q* < 0.05.

**Table 2:** Significantly changed resting state connectivity between IBS and HC females.

Resting-state connectivity	Beta	SE	*p* value	*t*	*q* value^a^	Change
Left PosDCgG to Left Pu	0.129509684	0.029287569	2.01405E−05	4.422001763	0.049184611	Increase in IBS
Right SupFG to Right Pu	0.133066023	0.030322822	2.30643E−05	4.388312563	0.049184611	Increase in IBS

*PosDCgG* dorsal part of the posterior cingulate gyrus, *SupFG* superior frontal gyrus, *Pu* putamen.

^a^Differences in resting-state connectivity between two groups were assessed by Student’s *t* test, controlling for age, BMI, and diet. *q* value was calculated with FDR correction.

### Significant correlation of metabolites and resting-state connectivity between IBS and HC females

**Fig. 2:**
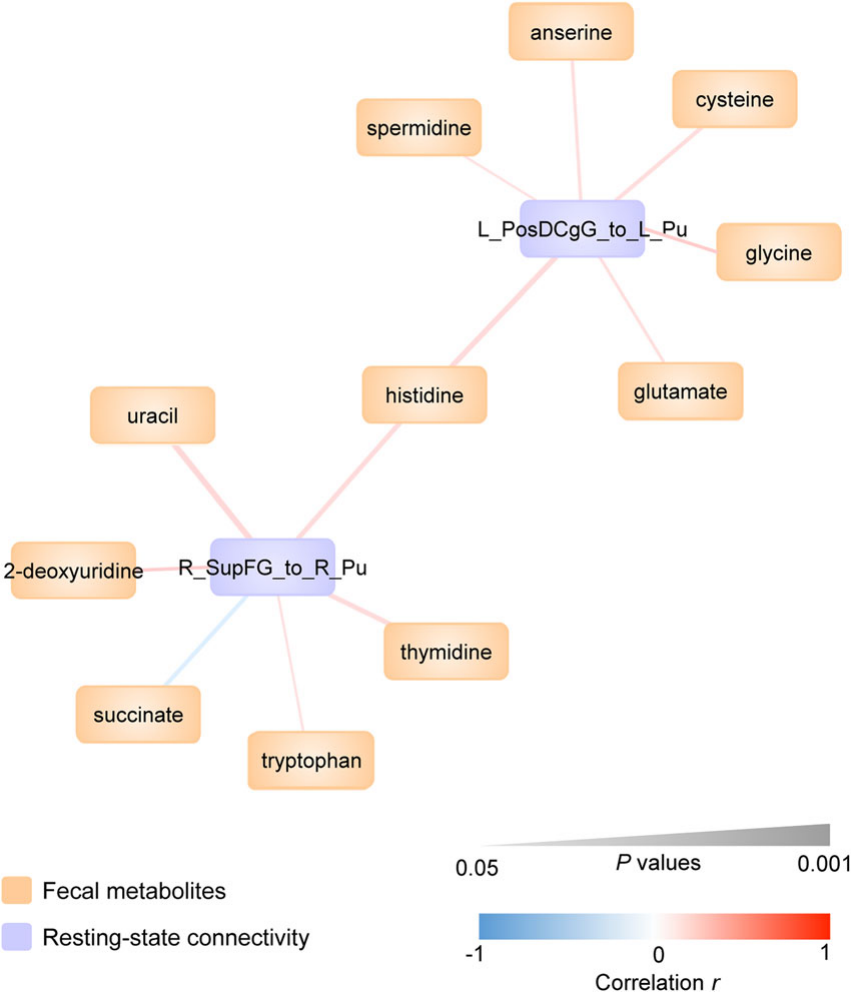
The partial correlation of significantly changed metabolites and resting-state connectivity controlling for age, BMI, and diet between IBS and HC females. L_PosDCgG dorsal part of the left posterior cingulate gyrus, L_Pu left putamen, R_Pu right putamen, R_SupFG right superior frontal gyrus.

The partial correlations of significantly changed metabolites and RS connectivity between IBS and HCs, corrected for age, BMI, and diet are shown in Table 3 and Fig. 2. In particular, the differences in histidine, cysteine, glycine, glutamate, spermidine, and anserine were significantly associated with the alteration in left dorsal part of the posterior cingulate gyrus to the left putamen (*p* < 0.05). In addition, the changes in histidine, tryptophan, uracil, 2-deoxyuridine, thymidine, and succinate were differentially associated with the alteration in the right superior frontal gyrus to right putamen (*p* < 0.05).

**Table 3:** Significant correlations of metabolites and RS connectivity between IBS and HC females.

Metabolites	RS connectivity	*r*	*p* value^a^
Histidine	Left PosDCgG to Left Pu	0.21	0.016
Cysteine	Left PosDCgG to Left Pu	0.19	0.030
Glycine	Left PosDCgG to Left Pu	0.17	0.043
Glutamate	Left PosDCgG to Left Pu	0.18	0.042
Spermidine	Left PosDCgG to Left Pu	0.17	0.050
Anserine	Left PosDCgG to Left Pu	0.18	0.040
Histidine	Right SupFG to Right Pu	0.20	0.022
Tryptophan	Right SupFG to Right Pu	0.17	0.050
Uracil	Right SupFG to Right Pu	0.22	0.010
2-Deoxyuridine	Right SupFG to Right Pu	0.19	0.027
Thymidine	Right SupFG to Right Pu	0.20	0.022
Succinate	Right SupFG to Right Pu	−0.19	0.029

*PosDCgG* dorsal part of the posterior cingulate gyrus, *SupFG* superior frontal gyrus, *Pu* putamen.

^a^Partial correlation analysis between metabolites and RS connectivity controlling for age, BMI, and diet was performed.

## Discussion

In this study, we demonstrate key differences in brain connectivity and fecal metabolites between premenauposal IBS and HC subjects and these findings were integrated to create a brain–gut interactome map. This interactome likely represents the result of both brain to gut and gut to brain processes that are, at least in part, influenced by the gut microbiome. While not providing information about causality, our findings are an important foundation for future mechanistic studies.

IBS females showed higher connectivity between the putamen (basal ganglia) and a region in the DMN (dorsal part of the posterior cingulate gyrus) and somatosensory network (superior frontal gyrus). This is consistent with previous work, which demonstrated topological reorganization of the DMN^26^ and widespread microstructural white matter changes of the somatosensory network^27^ in IBS. Additionally, past studies using experimental rectal stimulation suggest that the basal ganglia play a key role in modulating IBS-related alterations in pain processing^28,29^.

Existing evidence from deep brain stimulation reveals that the basal ganglia are involved in IBS symptom generation^30^. We have also shown that the putamen is a key brain region through which the gut microbiota may impact to increase visceral hypersensitivity in IBS. In a recently published study integrating experimental rectal balloon distension, 16S rRNA gene sequencing, and functional neuroimaging, *Clostridium XIVa* demonstrated an association between rectal discomfort, rectal pain, and oral–anal-transit time, which was linked to altered connectivity of the putamen^6^. Furthermore, a previous study which investigated the relationship between the gut microbiome and brain volumes also showed an association between Firmicutes-associated Clostridia, including *Clostridium XIVa*, and increased gray matter volume of the putamen in IBS patients^31^. When viewed together, the current findings confirm an important role of the putamen in altered visceral perception in IBS.

Analysis of fecal metabolites revealed a diverse group of metabolites and metabolic pathways that successfully differentiate IBS from HCs. Notable pathways include those involved in nucleic acid (tRNA biosynthesis, pyrimidine metabolism) and amino acid (alanine, aspartate, and glutamate metabolism) metabolism. In contrast to previous human investigations^32^, which employed a very targeted approach and analyzed, for example, 19 discrete fecal metabolites, we interrogated 51 pathways and over 1000 metabolites, to allow for novel hypothesis generation.

In generating the brain–gut interactome map, we see that only a subset of metabolites—primarily those involving in amino acid metabolism—were associated with IBS-specific brain changes. The selected metabolites that emerged in the interactome map may represent key drivers of IBS brain–gut pathophysiology. In contrast, metabolites that differentiate IBS from HC females but that did not appear in the interactome might represent the result of secondary, brain to gut influences on the gut microenvironment that do not impact the brain or play a key role in IBS development.

The essential amino acid histidine emerged as the only metabolite that was positively associated with all IBS-specific changes in functional connectivity. Histidine may interact with the brain directly, by crossing the blood–brain-barrier^33^ or indirectly through conversion into histamine by either host or bacterial histidine decarboxylase enzymes^34^. The gut microbiome may also modulate these interactions, as one in vitro study demonstrated that histidine is extensively modified and digested by gut microbes of the small intestine^35^. This host–microbe cross-talk, however, extends beyond modifying gut metabolites directly related to histidine/histamine, as some bacteria such as *Lactobacillus rhamnosus* can modulate the immune system through direct activation of histamine receptors^36^. Changes in histidine/histamine may contribute to IBS symptomatology, as one randomized controlled trial comparing the effects of a diet low in fermentable oligosaccharides, disaccharides, monosaccharides, and polyols (FODMAPs) and high in FODMAPs demonstrated reduction in IBS symptom severity scores and a greater than eightfold reduction in urine histamine levels in the low FODMAP group^37^. Furthermore, the association between this histamine metabolite and connectivity involving the putamen has not previously been described in IBS, but it is perhaps unsurprising, given that high expression of the histamine G-protein-coupled H1R, H2R, and H3R receptors has been identified in the basal ganglia^38^. When viewed together, the current results are consistent with a growing literature implicating histidine or its metabolites in IBS symptoms.

The amino acid tryptophan showed a positive association with increased connectivity between a region in the DMN (dorsal part of the posterior cingulate gyrus) to the basal ganglia (putamen). Tryptophan and its metabolites have been extensively studied within the context of the BGM axis and especially in IBS^39^. We have previously shown that spore forming microorganisms known to modulate gut motility can stimulate the biosynthesis and release of serotonin from intestinal enterochromaffin cells^40^. These microbes also demonstrate positive associations with putamen connectivity in IBS patients, but negative associations with HCs^6^. Our results further support the hypothesis that this interaction may be mediated by aberrant tryptophan signaling in IBS patients.

Our results also demonstrate an association between metabolites involved in pyrimidine metabolism (i.e. thymidine) and aberrant brain connectivity. Relatively few studies have explored this pathway within the context of IBS; however, one study of 23 IBS children and 22 HCs demonstrated an association between IBS pain intensity and pain frequency with thymine, the precursor to thymidine^41^. Data from a longitudinal human gut microbiota study suggests that enhanced pyrimidine metabolism may be indicative of gut microbiota dysbiosis^42^, further implicating the gut microbiome as playing a causative role in IBS. Exploring the role of this previously unstudied pathway in IBS may reveal new insights in how we understand this disease.

This study focused on fecal metabolites and functional connectivity of brain regions that have previously been implicated in IBS pathophysiology. Future work may benefit from incorporating clinical measures of IBS severity and alternative neuroimaging modalities, such as diffusion tensor imaging to also characterize differences in structural connectivity between IBS and HCs. Female patients with IBS are known to feel more fatigue, symptoms of depression, and report lower quality of life than men with IBS^5^, with high-quality data showing sex-differences in the neuroimaging findings of IBS^2^. Future studies would benefit from performing similar investigations in males with IBS. Although our discussion included the role of the gut microbiome in the interaction network between the described fecal metabolites and brain regions, we did not explicitly examine 16S rRNA gene-sequencing data in this study. Incorporating microbiota data into future analyses may provide important context for future investigations.

The directionality and causality between fecal metabolites and alterations in brain connectivity cannot be parsed, though previous work has suggested a bidirectional model for BGM communication in IBS^43^. In the absence of a truly valid IBS animal model and the lack of effective treatments targeting specific targets within the BGM axis which could be used as probes, and the challenges of doing studies in humans to address the bidirectional BGM interactions, cross-sectional studies such as the one we present here are a crucial step in identifying BGM interactions that may be driving IBS pathophysiology.

To our knowledge, this is the first study to integrate functional neuroimaging and fecal metabolite data to create an integrated BGM model of IBS. While supporting previous preclinical and clinical work in this patient population, the study revealed novel insights, which are essential to provide the foundation for previously unexplored avenues in understanding IBS pathophysiology, particularly with respect to how the gut microbiota participates in the complex cross-talk between gut metabolites and aberrant brain connectivity.

## Supplementary information

Supplemental Table S1

Supplemental Table S2
