# Supplementary material for: Analysis of brain networks and fecal metabolites reveals brain–gut alterations in premenopausal females with irritable bowel syndrome
Source: Transl Psychiatry. 2020 Nov 2;10:367. doi: 10.1038/s41398-020-01071-2 (PMC7608552; doi:10.1038/s41398-020-01071-2)
Supplement: Supplementary file 1 — Supplemental Table S1 [file 41398_2020_1071_MOESM1_ESM.docx]

**Table S1. Significantly changed VIP metabolites between IBS and HC females**

| **Significantly changed VIP metabolites (IBS vs HC)** | **Super Pathway** | **Sub Pathway** | **beta** | **se** | ***P* value** | **t** | **Fold Change** | ***q* value^1^** |
| --- | --- | --- | --- | --- | --- | --- | --- | --- |
| alanine | Amino Acid | Alanine and Aspartate Metabolism | 0.79 | 0.18 | 1.29048E-05 | 4.53 | 1.29 | 8.2591E-05 |
| N-acetylalanine | Amino Acid | Alanine and Aspartate Metabolism | -0.95 | 0.16 | 1.22314E-08 | -6.07 | 0.40 | 1.95702E-07 |
| N-acetylasparagine | Amino Acid | Alanine and Aspartate Metabolism | 0.47 | 0.18 | 0.011465207 | 2.56 | 0.74 | 0.015857406 |
| glutamate | Amino Acid | Glutamate Metabolism | 0.61 | 0.18 | 0.001025293 | 3.36 | 1.22 | 0.001940626 |
| N-methyl-GABA | Amino Acid | Glutamate Metabolism | 0.41 | 0.19 | 0.030842045 | 2.18 | 1.60 | 0.031836949 |
| 5-oxoproline | Amino Acid | Glutathione Metabolism | 0.60 | 0.18 | 0.001275819 | 3.29 | 1.42 | 0.002268123 |
| 2-methylserine | Amino Acid | Glycine, Serine and Threonine Metabolism | -0.39 | 0.19 | 0.036859351 | -2.11 | 0.65 | 0.036859351 |
| glycine | Amino Acid | Glycine, Serine and Threonine Metabolism | 0.64 | 0.18 | 0.000541366 | 3.54 | 1.28 | 0.001332594 |
| N-acetylserine | Amino Acid | Glycine, Serine and Threonine Metabolism | -0.60 | 0.18 | 0.001021751 | -3.36 | 0.58 | 0.001940626 |
| serine | Amino Acid | Glycine, Serine and Threonine Metabolism | 0.44 | 0.19 | 0.020172794 | 2.35 | 1.13 | 0.021517647 |
| anserine | Amino Acid | Histidine Metabolism | 0.67 | 0.18 | 0.000344092 | 3.67 | 1.34 | 0.000917579 |
| histidine | Amino Acid | Histidine Metabolism | 0.63 | 0.18 | 0.000674502 | 3.48 | 1.30 | 0.00154172 |
| N-acetylhistamine | Amino Acid | Histidine Metabolism | 0.62 | 0.18 | 0.001030958 | 3.36 | 1.70 | 0.001940626 |
| 2,3-dimethylsuccinate | Amino Acid | Leucine, Isoleucine and Valine Metabolism | -0.48 | 0.19 | 0.011893055 | -2.55 | 0.56 | 0.015857406 |
| 3-methyl-2-oxovalerate | Amino Acid | Leucine, Isoleucine and Valine Metabolism | 0.68 | 0.18 | 0.000201644 | 3.82 | 1.49 | 0.000645259 |
| 4-methyl-2-oxopentanoate | Amino Acid | Leucine, Isoleucine and Valine Metabolism | 0.68 | 0.18 | 0.000186833 | 3.84 | 1.45 | 0.000645259 |
| isoleucine | Amino Acid | Leucine, Isoleucine and Valine Metabolism | 0.44 | 0.19 | 0.019670164 | 2.36 | 1.15 | 0.021517647 |
| leucine | Amino Acid | Leucine, Isoleucine and Valine Metabolism | 0.51 | 0.19 | 0.0071964 | 2.73 | 1.16 | 0.010965943 |
| 2-aminoadipate | Amino Acid | Lysine Metabolism | 0.91 | 0.17 | 5.56173E-07 | 5.26 | 1.48 | 5.93252E-06 |
| 6-oxopiperidine-2-carboxylate | Amino Acid | Lysine Metabolism | -0.77 | 0.18 | 2.14084E-05 | -4.41 | 0.48 | 0.000114178 |
| cysteine | Amino Acid | Methionine, Cysteine, SAM and Taurine Metabolism | 0.46 | 0.19 | 0.013688717 | 2.50 | 1.15 | 0.016847651 |
| methionine | Amino Acid | Methionine, Cysteine, SAM and Taurine Metabolism | 0.68 | 0.18 | 0.000242923 | 3.77 | 1.24 | 0.000706685 |
| N-acetylmethionine sulfoxide | Amino Acid | Methionine, Cysteine, SAM and Taurine Metabolism | 1.04 | 0.16 | 2.23998E-09 | 6.41 | 1.73 | 7.16794E-08 |
| spermidine | Amino Acid | Polyamine Metabolism | 0.50 | 0.19 | 0.009301846 | 2.64 | 1.07 | 0.013529957 |
| indoleacetate | Amino Acid | Tryptophan Metabolism | 0.46 | 0.19 | 0.01509698 | 2.46 | 1.08 | 0.017253691 |
| tryptophan | Amino Acid | Tryptophan Metabolism | 0.52 | 0.19 | 0.005761248 | 2.81 | 1.16 | 0.009217996 |
| gentisate | Amino Acid | Tyrosine Metabolism | -0.72 | 0.18 | 9.79042E-05 | -4.02 | 0.35 | 0.000447562 |
| N-acetyltyrosine | Amino Acid | Tyrosine Metabolism | 0.47 | 0.19 | 0.014303787 | 2.48 | 1.32 | 0.016952637 |
| tyrosine | Amino Acid | Tyrosine Metabolism | 0.54 | 0.19 | 0.003968884 | 2.93 | 1.18 | 0.006684436 |
| citrulline | Amino Acid | Urea cycle; Arginine and Proline Metabolism | 0.47 | 0.19 | 0.012643358 | 2.53 | 1.15 | 0.016183498 |
| homocitrulline | Amino Acid | Urea cycle; Arginine and Proline Metabolism | 0.89 | 0.17 | 7.77939E-07 | 5.19 | 1.58 | 6.22351E-06 |
| N-delta-acetylornithine | Amino Acid | Urea cycle; Arginine and Proline Metabolism | 0.69 | 0.18 | 0.00015378 | 3.90 | 1.26 | 0.00061512 |
| N6-carboxymethyllysine | Carbohydrate | Advanced Glycation End-product | -0.41 | 0.19 | 0.028957698 | -2.21 | 0.75 | 0.028957698 |
| erythronate | Carbohydrate | Aminosugar Metabolism | -0.90 | 0.17 | 4.82555E-07 | -5.29 | 0.26 | 3.61917E-06 |
| N-acetyl-beta-glucosaminylamine | Carbohydrate | Aminosugar Metabolism | 0.54 | 0.18 | 0.003906198 | 2.94 | 1.43 | 0.005326633 |
| N-acetylglucosamine/N-acetylgalactosamine | Carbohydrate | Aminosugar Metabolism | 0.71 | 0.18 | 9.34619E-05 | 4.03 | 1.46 | 0.000350482 |
| N-acetylglucosaminylasparagine | Carbohydrate | Aminosugar Metabolism | 0.56 | 0.19 | 0.003172743 | 3.00 | 1.52 | 0.004937651 |
| N-acetylmuramate | Carbohydrate | Aminosugar Metabolism | 0.47 | 0.19 | 0.013130997 | 2.51 | 1.45 | 0.014068926 |
| N-acetylneuraminate | Carbohydrate | Aminosugar Metabolism | 0.53 | 0.18 | 0.004537913 | 2.89 | 1.34 | 0.005672391 |
| fructose | Carbohydrate | Fructose, Mannose and Galactose Metabolism | -0.49 | 0.18 | 0.006857335 | -2.75 | 0.54 | 0.007912309 |
| galactonate | Carbohydrate | Fructose, Mannose and Galactose Metabolism | -0.72 | 0.18 | 8.55377E-05 | -4.05 | 0.51 | 0.000350482 |
| mannitol/sorbitol | Carbohydrate | Fructose, Mannose and Galactose Metabolism | -0.65 | 0.17 | 0.00023118 | -3.78 | 0.27 | 0.00057795 |
| glycerate | Carbohydrate | Glycolysis, Gluconeogenesis, and Pyruvate Metabolism | -0.64 | 0.18 | 0.000464932 | -3.59 | 0.56 | 0.000996282 |
| arabonate/xylonate | Carbohydrate | Pentose Metabolism | -0.90 | 0.17 | 3.69065E-07 | -5.35 | 0.34 | 3.61917E-06 |
| fucose | Carbohydrate | Pentose Metabolism | 0.60 | 0.18 | 0.001310857 | 3.28 | 1.32 | 0.002457857 |
| ribonate | Carbohydrate | Pentose Metabolism | -0.54 | 0.18 | 0.003291768 | -2.99 | 0.37 | 0.004937651 |
| sedoheptulose | Carbohydrate | Pentose Metabolism | 0.71 | 0.18 | 0.000123776 | 3.95 | 1.41 | 0.000371329 |
| threonate | Cofactors and Vitamins | Ascorbate and Aldarate Metabolism | -0.87 | 0.17 | 1.67649E-06 | -5.01 | 0.31 | 4.19123E-06 |
| nicotinate ribonucleoside | Cofactors and Vitamins | Nicotinate and Nicotinamide Metabolism | -0.50 | 0.18 | 0.007399428 | -2.72 | 0.67 | 0.007399428 |
| pterin | Cofactors and Vitamins | Pterin Metabolism | 0.90 | 0.17 | 4.86327E-07 | 5.29 | 1.26 | 2.43164E-06 |
| alpha-tocopherol | Cofactors and Vitamins | Tocopherol Metabolism | 0.51 | 0.18 | 0.00554892 | 2.82 | 1.29 | 0.006936151 |
| gamma-tocotrienol | Cofactors and Vitamins | Tocopherol Metabolism | 0.61 | 0.18 | 0.001249391 | 3.30 | 1.48 | 0.002082318 |
| alpha-ketoglutarate | Energy | TCA Cycle | 0.66 | 0.18 | 0.000316746 | 3.70 | 1.73 | 0.000633492 |
| succinate | Energy | TCA Cycle | -0.48 | 0.19 | 0.011476527 | -2.56 | 0.46 | 0.011476527 |
| ceramide (d18:1/14:0, d16:1/16:0) | Lipid | Ceramides | -0.54 | 0.18 | 0.003646869 | -2.96 | 0.63 | 0.005801837 |
| N-palmitoyl-sphingosine (d18:1/16:0) | Lipid | Ceramides | -0.47 | 0.18 | 0.011665156 | -2.56 | 0.66 | 0.014581445 |
| diacylglycerol (16:1/18:2 [2], 16:0/18:3 [1]) | Lipid | Diacylglycerol | -0.73 | 0.18 | 6.24976E-05 | -4.13 | 0.33 | 0.000156244 |
| linoleoyl-linoleoyl-glycerol (18:2/18:2) [1] | Lipid | Diacylglycerol | -0.41 | 0.19 | 0.030722186 | -2.18 | 0.41 | 0.032331753 |
| linoleoyl-linoleoyl-glycerol (18:2/18:2) [2] | Lipid | Diacylglycerol | -0.57 | 0.19 | 0.002724095 | -3.05 | 0.52 | 0.004540158 |
| oleoyl-linoleoyl-glycerol (18:1/18:2) [1] | Lipid | Diacylglycerol | -0.39 | 0.19 | 0.03773058 | -2.10 | 0.32 | 0.03773058 |
| oleoyl-linoleoyl-glycerol (18:1/18:2) [2] | Lipid | Diacylglycerol | -0.41 | 0.19 | 0.029110339 | -2.21 | 0.43 | 0.032331753 |
| oleoyl-oleoyl-glycerol (18:1/18:1) [1] | Lipid | Diacylglycerol | -0.95 | 0.17 | 1.01595E-07 | -5.63 | 0.19 | 7.11167E-07 |
| oleoyl-oleoyl-glycerol (18:1/18:1) [2] | Lipid | Diacylglycerol | -0.58 | 0.18 | 0.001684408 | -3.21 | 0.31 | 0.003102857 |
| palmitoyl-linoleoyl-glycerol (16:0/18:2) [1] | Lipid | Diacylglycerol | -0.78 | 0.17 | 1.69328E-05 | -4.46 | 0.21 | 4.93872E-05 |
| palmitoyl-linoleoyl-glycerol (16:0/18:2) [2] | Lipid | Diacylglycerol | -0.69 | 0.18 | 0.000173087 | -3.86 | 0.29 | 0.000403869 |
| palmitoyl-oleoyl-glycerol (16:0/18:1) [2] | Lipid | Diacylglycerol | -1.00 | 0.16 | 9.33101E-09 | -6.13 | 0.12 | 1.08862E-07 |
| N-palmitoyl-sphinganine (d18:0/16:0) | Lipid | Dihydroceramides | -1.09 | 0.16 | 9.76734E-11 | -7.03 | 0.14 | 1.70928E-09 |
| linoleoyl ethanolamide | Lipid | Endocannabinoid | 0.41 | 0.19 | 0.031407989 | 2.17 | 3.31 | 0.032331753 |
| LAHSA (18:2/OH-18:0) | Lipid | Fatty Acid Hydroxyl Fatty Acid | -0.44 | 0.18 | 0.019392053 | -2.37 | 0.59 | 0.022624062 |
| malonate | Lipid | Fatty Acid Synthesis | 0.91 | 0.17 | 5.81426E-07 | 5.25 | 1.63 | 2.90713E-06 |
| 2-hydroxyglutarate | Lipid | Fatty Acid, Dicarboxylate | 1.11 | 0.15 | 3.53431E-11 | 7.22 | 1.47 | 1.23701E-09 |
| adipate | Lipid | Fatty Acid, Dicarboxylate | -0.41 | 0.19 | 0.030783148 | -2.18 | 0.40 | 0.032331753 |
| maleate | Lipid | Fatty Acid, Dicarboxylate | -0.60 | 0.19 | 0.001445232 | -3.25 | 0.64 | 0.002810173 |
| 13-HODE + 9-HODE | Lipid | Fatty Acid, Monohydroxy | 0.81 | 0.17 | 7.23212E-06 | 4.67 | 1.58 | 2.53124E-05 |
| 2-hydroxybehenate | Lipid | Fatty Acid, Monohydroxy | 0.61 | 0.18 | 0.001086623 | 3.34 | 1.64 | 0.002237164 |
| 2-hydroxypalmitate | Lipid | Fatty Acid, Monohydroxy | 0.46 | 0.19 | 0.013496712 | 2.50 | 1.16 | 0.016289136 |
| 10-heptadecenoate (17:1n7) | Lipid | Long Chain Fatty Acid | 0.50 | 0.19 | 0.007540009 | 2.71 | 1.32 | 0.010556013 |
| nervonate (24:1n9) | Lipid | Long Chain Monounsaturated Fatty Acid | 0.97 | 0.16 | 3.31998E-08 | 5.87 | 1.90 | 2.90499E-07 |
| palmitoleate (16:1n7) | Lipid | Long Chain Monounsaturated Fatty Acid | 0.49 | 0.19 | 0.010382599 | 2.60 | 1.24 | 0.013529967 |
| arachidate (20:0) | Lipid | Long Chain Saturated Fatty Acid | 0.74 | 0.17 | 2.90241E-05 | 4.33 | 1.52 | 7.81417E-05 |
| behenate (22:0) | Lipid | Long Chain Saturated Fatty Acid | 0.60 | 0.18 | 0.001024906 | 3.36 | 1.48 | 0.002237164 |
| margarate (17:0) | Lipid | Long Chain Saturated Fatty Acid | 0.87 | 0.17 | 6.74484E-07 | 5.22 | 1.75 | 2.95087E-06 |
| nonadecanoate (19:0) | Lipid | Long Chain Saturated Fatty Acid | 0.78 | 0.17 | 1.04865E-05 | 4.58 | 1.70 | 3.33661E-05 |
| palmitate (16:0) | Lipid | Long Chain Saturated Fatty Acid | 0.83 | 0.17 | 2.27721E-06 | 4.94 | 1.47 | 8.85583E-06 |
| stearate (18:0) | Lipid | Long Chain Saturated Fatty Acid | 0.91 | 0.16 | 1.75327E-07 | 5.51 | 1.61 | 1.02274E-06 |
| 1-palmitoyl-GPE (16:0) | Lipid | Lysophospholipid | -0.57 | 0.18 | 0.002051289 | -3.14 | 0.64 | 0.003589755 |
| mevalonate | Lipid | Mevalonate Metabolism | -0.53 | 0.18 | 0.00398686 | -2.93 | 0.38 | 0.006066961 |
| phytosphingosine | Lipid | Sphingolipid Synthesis | -0.48 | 0.19 | 0.010437403 | -2.60 | 0.51 | 0.013529967 |
| heptadecasphingosine (d17:1) | Lipid | Sphingosines | -0.51 | 0.19 | 0.007285069 | -2.73 | 0.26 | 0.010556013 |
| 2'-deoxyinosine | Nucleotide | Purine Metabolism, (Hypo)Xanthine/Inosine containing | 0.51 | 0.19 | 0.007473059 | 2.72 | 1.27 | 0.009772462 |
| hypoxanthine | Nucleotide | Purine Metabolism, (Hypo)Xanthine/Inosine containing | 0.51 | 0.19 | 0.007046358 | 2.74 | 1.26 | 0.009772462 |
| inosine | Nucleotide | Purine Metabolism, (Hypo)Xanthine/Inosine containing | 0.84 | 0.18 | 6.08497E-06 | 4.71 | 1.81 | 3.44815E-05 |
| urate | Nucleotide | Purine Metabolism, (Hypo)Xanthine/Inosine containing | 0.46 | 0.19 | 0.014022775 | 2.49 | 1.38 | 0.015892479 |
| xanthine | Nucleotide | Purine Metabolism, (Hypo)Xanthine/Inosine containing | 0.59 | 0.18 | 0.001511044 | 3.24 | 1.30 | 0.003669678 |
| 1-methyladenine | Nucleotide | Purine Metabolism, Adenine containing | 0.49 | 0.19 | 0.009227254 | 2.64 | 1.19 | 0.011204523 |
| 7-methylguanine | Nucleotide | Purine Metabolism, Guanine containing | 0.92 | 0.17 | 3.0121E-07 | 5.39 | 1.31 | 5.12058E-06 |
| guanine | Nucleotide | Purine Metabolism, Guanine containing | 0.46 | 0.19 | 0.015114269 | 2.46 | 1.18 | 0.016058911 |
| 2'-deoxycytidine | Nucleotide | Pyrimidine Metabolism, Cytidine containing | 0.54 | 0.19 | 0.004780664 | 2.87 | 1.49 | 0.007488926 |
| N-carbamoylaspartate | Nucleotide | Pyrimidine Metabolism, Orotate containing | 0.64 | 0.18 | 0.000415212 | 3.62 | 1.93 | 0.001613883 |
| thymidine | Nucleotide | Pyrimidine Metabolism, Thymine containing | 0.54 | 0.19 | 0.004845776 | 2.86 | 1.17 | 0.007488926 |
| thymine | Nucleotide | Pyrimidine Metabolism, Thymine containing | 0.42 | 0.19 | 0.025332764 | 2.26 | 1.16 | 0.025332764 |
| 2'-deoxyuridine | Nucleotide | Pyrimidine Metabolism, Uracil containing | 0.88 | 0.18 | 1.7257E-06 | 5.00 | 1.44 | 1.46684E-05 |
| 5-methyluridine (ribothymidine) | Nucleotide | Pyrimidine Metabolism, Uracil containing | 0.57 | 0.19 | 0.002903195 | 3.03 | 1.38 | 0.005483813 |
| pseudouridine | Nucleotide | Pyrimidine Metabolism, Uracil containing | 0.65 | 0.18 | 0.000481139 | 3.58 | 1.55 | 0.001613883 |
| uracil | Nucleotide | Pyrimidine Metabolism, Uracil containing | 0.58 | 0.18 | 0.002041665 | 3.15 | 1.32 | 0.004338538 |
| uridine | Nucleotide | Pyrimidine Metabolism, Uracil containing | 0.65 | 0.18 | 0.000569606 | 3.53 | 1.31 | 0.001613883 |
| glutaminylleucine | Peptide | Dipeptide | 0.54 | 0.19 | 0.004140283 | 2.92 | 1.22 | 0.006207391 |
| glycylisoleucine | Peptide | Dipeptide | 0.60 | 0.19 | 0.001626047 | 3.22 | 1.23 | 0.003794109 |
| glycylvaline | Peptide | Dipeptide | 0.48 | 0.19 | 0.011122501 | 2.57 | 1.15 | 0.011122501 |
| isoleucylglycine | Peptide | Dipeptide | 0.87 | 0.17 | 1.56842E-06 | 5.03 | 1.61 | 5.48947E-06 |
| gamma-glutamylglutamate | Peptide | Gamma-glutamyl Amino Acid | 0.54 | 0.19 | 0.00443385 | 2.89 | 1.22 | 0.006207391 |
| gamma-glutamylisoleucine | Peptide | Gamma-glutamyl Amino Acid | 0.49 | 0.19 | 0.008854969 | 2.66 | 1.23 | 0.010330797 |
| gamma-glutamylleucine | Peptide | Gamma-glutamyl Amino Acid | 1.15 | 0.15 | 1.20581E-11 | 7.42 | 1.57 | 8.44067E-11 |
| 4-hydroxybenzoate | Xenobiotics | Benzoate Metabolism | 0.50 | 0.19 | 0.007898259 | 2.70 | 1.25 | 0.009477911 |
| benzoate | Xenobiotics | Benzoate Metabolism | 0.57 | 0.19 | 0.002422192 | 3.09 | 1.22 | 0.004844385 |
| p-cresol sulfate | Xenobiotics | Benzoate Metabolism | 0.57 | 0.19 | 0.002365337 | 3.10 | 1.30 | 0.004844385 |
| p-hydroxybenzaldehyde | Xenobiotics | Benzoate Metabolism | 0.72 | 0.18 | 7.81393E-05 | 4.08 | 1.40 | 0.000468836 |
| 2-isopropylmalate | Xenobiotics | Food Component/Plant | -0.50 | 0.18 | 0.006345895 | -2.77 | 0.34 | 0.009477911 |
| 2-keto-3-deoxy-gluconate | Xenobiotics | Food Component/Plant | -0.68 | 0.18 | 0.000246441 | -3.77 | 0.54 | 0.000985765 |
| 4-hydroxycinnamate | Xenobiotics | Food Component/Plant | -0.77 | 0.18 | 3.6344E-05 | -4.27 | 0.31 | 0.000436127 |
| diaminopimelate | Xenobiotics | Food Component/Plant | 0.45 | 0.19 | 0.019247273 | 2.37 | 1.50 | 0.019247273 |
| gluconate | Xenobiotics | Food Component/Plant | -0.53 | 0.18 | 0.003913558 | -2.94 | 0.52 | 0.006708956 |
| pheophorbide A | Xenobiotics | Food Component/Plant | -0.50 | 0.19 | 0.007820436 | -2.70 | 0.39 | 0.009477911 |
| phytanate | Xenobiotics | Food Component/Plant | 0.60 | 0.18 | 0.001010249 | 3.36 | 1.66 | 0.003030748 |
| 5-acetylamino-6-amino-3-methyluracil | Xenobiotics | Xanthine Metabolism | -0.48 | 0.19 | 0.010128151 | -2.61 | 0.39 | 0.011048892 |

^1^ Differences in VIP metabolites between two groups were assessed by Student’s *t* test, controlling for Age, BMI, and Diet. *P* value was calculated with FDR correction.
