# Supplementary material for: Analysis of brain networks and fecal metabolites reveals brain–gut alterations in premenopausal females with irritable bowel syndrome
Source: Transl Psychiatry. 2020 Nov 2;10:367. doi: 10.1038/s41398-020-01071-2 (PMC7608552; doi:10.1038/s41398-020-01071-2)
Supplement: Supplementary file 2 — Supplemental Table S2 [file 41398_2020_1071_MOESM2_ESM.docx]

**Table S2. Total identified pathways in KEGG between HC and IBS females**

| **Pathways** | **Total** | **Expected** | **Hits** |
| --- | --- | --- | --- |
| Aminoacyl-tRNA biosynthesis | 75 | 2.65 | 11 |
| Pyrimidine metabolism | 60 | 2.12 | 9 |
| Alanine, aspartate and glutamate metabolism | 24 | 0.85 | 5 |
| beta-Alanine metabolism | 28 | 0.99 | 5 |
| Glutathione metabolism | 38 | 1.34 | 5 |
| Nitrogen metabolism | 39 | 1.38 | 5 |
| Valine, leucine and isoleucine biosynthesis | 27 | 0.95 | 4 |
| Histidine metabolism | 44 | 1.55 | 5 |
| Phenylalanine metabolism | 45 | 1.59 | 5 |
| Glycine, serine and threonine metabolism | 48 | 1.70 | 5 |
| Ubiquinone and other terpenoid-quinone biosynthesis | 36 | 1.27 | 4 |
| Purine metabolism | 92 | 3.25 | 7 |
| Butanoate metabolism | 40 | 1.41 | 4 |
| Thiamine metabolism | 24 | 0.85 | 3 |
| D-Glutamine and D-glutamate metabolism | 11 | 0.39 | 2 |
| Sphingolipid metabolism | 25 | 0.88 | 3 |
| Lysine biosynthesis | 32 | 1.13 | 3 |
| Pentose phosphate pathway | 32 | 1.13 | 3 |
| Cyanoamino acid metabolism | 16 | 0.57 | 2 |
| Sulfur metabolism | 18 | 0.64 | 2 |
| Cysteine and methionine metabolism | 56 | 1.98 | 4 |
| Taurine and hypotaurine metabolism | 20 | 0.71 | 2 |
| Citrate cycle (TCA cycle) | 20 | 0.71 | 2 |
| Valine, leucine and isoleucine degradation | 40 | 1.41 | 3 |
| Caffeine metabolism | 21 | 0.74 | 2 |
| Pantothenate and CoA biosynthesis | 27 | 0.95 | 2 |
| Phenylalanine, tyrosine and tryptophan biosynthesis | 27 | 0.95 | 2 |
| Fatty acid biosynthesis | 49 | 1.73 | 3 |
| Glyoxylate and dicarboxylate metabolism | 50 | 1.77 | 3 |
| Arginine and proline metabolism | 77 | 2.72 | 4 |
| Methane metabolism | 34 | 1.20 | 2 |
| Nicotinate and nicotinamide metabolism | 44 | 1.55 | 2 |
| Ascorbate and aldarate metabolism | 45 | 1.59 | 2 |
| Lysine degradation | 47 | 1.66 | 2 |
| Tyrosine metabolism | 76 | 2.68 | 3 |
| Selenoamino acid metabolism | 22 | 0.78 | 1 |
| Fatty acid elongation in mitochondria | 27 | 0.95 | 1 |
| Pyruvate metabolism | 32 | 1.13 | 1 |
| Vitamin B6 metabolism | 32 | 1.13 | 1 |
| Glycerolipid metabolism | 32 | 1.13 | 1 |
| Terpenoid backbone biosynthesis | 33 | 1.17 | 1 |
| Propanoate metabolism | 35 | 1.24 | 1 |
| Galactose metabolism | 41 | 1.45 | 1 |
| Tryptophan metabolism | 79 | 2.79 | 2 |
| Primary bile acid biosynthesis | 47 | 1.66 | 1 |
| Fructose and mannose metabolism | 48 | 1.70 | 1 |
| Amino sugar and nucleotide sugar metabolism | 88 | 3.11 | 2 |
| Starch and sucrose metabolism | 50 | 1.77 | 1 |
| Fatty acid metabolism | 50 | 1.77 | 1 |
| Pentose and glucuronate interconversions | 53 | 1.87 | 1 |
| Porphyrin and chlorophyll metabolism | 104 | 3.67 | 2 |
